# Supplementary material for: Nuclear hormone receptor NHR-49 acts in parallel with HIF-1 to promote hypoxia adaptation in Caenorhabditis elegans
Source: eLife. 2022 Mar 14;11:e67911. doi: 10.7554/eLife.67911 (PMC8959602; doi:10.7554/eLife.67911)
Supplement: Supplementary file 1. — Statistical comparison of each genotype’s ability to reach at least L4 following 24 hr exposure to 0.5% O2 as embryo and then allowed to recover at 21% O2 for 65 hr compared to worm embryos kept in 21% O2 for 65 hr (two-way ANOVA corrected for multiple comparisons using the Tukey method). [file elife-67911-supp1.docx]

**Supplementary File 1.** **Statistical comparison of each genotype’s ability to reach at least L4 following 24 hr exposure to 0.5% O_2_ as embryo and then allowed to recover at 21% O_2_ for 65 hr, compared to worm embryos kept in 21% O_2_ for 65 hr (two-way ANOVA corrected for multiple comparisons using the Tukey method).**

| **0.5% O_2_ Figure** | **21% O_2_ Figure** | **Genotype** | **p-value** |
| --- | --- | --- | --- |
| Figure 2A | Figure 2—figure supplement 1A | WT | 0.4943 |
| Figure 2A | Figure 2—figure supplement 1A | *nhr-49(nr2041)* | <0.0001**** |
| Figure 2A | Figure 2—figure supplement 1A | *hif-1(ia4)* | <0.0001**** |
| Figure 2A | Figure 2—figure supplement 1A | *nhr-49(nr2041);hif-1(ia4)* | <0.0001**** |
| Figure 3E | Figure 4—figure supplement 1A | WT | 0.1403 |
| Figure 3E | Figure 4—figure supplement 1A | *nhr-49(nr2041)* | <0.0001**** |
| Figure 3E | Figure 4—figure supplement 1A | *fmo-2(ok2147)* | 0.0005*** |
| Figure 3E | Figure 4—figure supplement 1A | *acs-2(ok2457)* | 0.0008*** |
| Figure 3E | Figure 4—figure supplement 1A | *fmo-2(ok2147);acs-2(ok2157)* | <0.0001**** |
| Figure 4E | Figure 4—figure supplement 1D | WT | 0.1833 |
| Figure 4E | Figure 4—figure supplement 1D | *nhr-49(nr2041)* | <0.0001**** |
| Figure 4E | Figure 4—figure supplement 1D | *lgg-2(tm5755)* | 0.0014** |
| Figure 4E | Figure 4—figure supplement 1D | *nhr-49(nr2041); lgg-2(tm5755)* | 0.0002*** |
| Figure 4F | Figure 4—figure supplement 1E | WT | 0.8413 |
| Figure 4F | Figure 4—figure supplement 1E | *nhr-49(nr2041)* | <0.0001**** |
| Figure 4F | Figure 4—figure supplement 1E | *epg-6(tm8366)* | <0.0001**** |
| Figure 4F | Figure 4—figure supplement 1E | *nhr-49(nr2041); epg-6(tm8366)* | <0.0001**** |
| Figure 4—figure supplement 1F | Figure 4—figure supplement 1G | WT;*EV(RNAi)* | 0.9995 |
| Figure 4—figure supplement 1F | Figure 4—figure supplement 1G | *nhr-49(nr2041);EV(RNAi)* | 0.0003*** |
| Figure 4—figure supplement 1F | Figure 4—figure supplement 1G | WT;*nhr-49(RNAi)* | 0.0001*** |
| Figure 4—figure supplement 1F | Figure 4—figure supplement 1G | *nhr-49(nr2041);nhr-49(RNAi)* | 0.0002*** |
| Figure 4—figure supplement 1F | Figure 4—figure supplement 1G | WT;*atg-10(RNAi)* | 0.0001*** |
| Figure 4—figure supplement 1F | Figure 4—figure supplement 1G | *nhr-49(nr2041);atg-10(RNAi)* | 0.0021** |
| Figure 4—figure supplement 1F | Figure 4—figure supplement 1G | WT;*atg-7(RNAi)* | 0.0003*** |
| Figure 4—figure supplement 1F | Figure 4—figure supplement 1G | *nhr-49(nr2041);atg-7(RNAi)* | 0.0534 |
| Figure 4—figure supplement 1G | Figure 4—figure supplement 1G | WT;*bec-1(RNAi)* | 0.0015** |
| Figure 4—figure supplement 1F | Figure 4—figure supplement 1G | *nhr-49(nr2041);bec-1(RNAi)* | 0.0033** |
| Figure 4—figure supplement 1F | Figure 4—figure supplement 1G | WT;*epg-3(RNAi)* | 0.0006*** |
| Figure 4—figure supplement 1F | Figure 4—figure supplement 1G | *nhr-49(nr2041);epg-3(RNAi)* | <0.0001*** |
| Figure 6—figure supplement 1D | Figure 6—figure supplement 1E | WT;*EV(RNAi)* | 0.7899 |
| Figure 6—figure supplement 1D | Figure 6—figure supplement 1E | WT; *nhr-67(RNAi)* | <0.0001*** |
| Figure 6—figure supplement 1D | Figure 6—figure supplement 1E | *nhr-49(nr2041); EV(RNAi)* | <0.0001*** |
| Figure 6—figure supplement 1D | Figure 6—figure supplement 1E | *nhr-49(nr2041);nhr-67(RNAi)* | <0.0001*** |
| Figure 6—figure supplement 1D | Figure 6—figure supplement 1E | *hif-1(ia4); EV(RNAi)* | <0.0001*** |
| Figure 6—figure supplement 1D | Figure 6—figure supplement 1E | *hif-1(ia4); nhr-67(RNAi)* | <0.0001*** |
| Figure 7F | Figure 7—figure supplement 1C | WT | 0.9119 |
| Figure 7F | Figure 7—figure supplement 1C | *nhr-49(nr2041)* | <0.0001**** |
| Figure 7F | Figure 7—figure supplement 1C | *hpk-1(pk1393)* | <0.0001**** |
| Figure 7F | Figure 7—figure supplement 1C | *nhr-49(nr2041);hpk-1(pk1393)* | <0.0001**** |
| Figure 7G | Figure 7—figure supplement 1D | WT | 0.0223* |
| Figure 7G | Figure 7—figure supplement 1D | *hif-1(ia4)* | <0.0001**** |
| Figure 7G | Figure 7—figure supplement 1D | *hpk-1(pk1393)* | <0.0001**** |
| Figure 7G | Figure 7—figure supplement 1D | *hif-1(ia4);hpk-1(pk1393)* | <0.0001**** |

All p-values are derived using ordinary two-way ANOVA corrected for multiple comparisons using the Tukey method. *p<0.05, **p<0.01, ***p<0.001, and ****p<0.0001. WT = wild-type.

**Supplementary File 2.** **Statistical comparison of each genotype’s ability to reach at least L4 stage from L1 stage following 48 hr exposure to 0.5% O_2_ as embryos, compared to animals kept in 21% O_2_ for 48 hr.**

| **0.5% O_2_ Figure** | **21% O_2_ Figure** | **Genotype** | **p-value** |
| --- | --- | --- | --- |
| Figure 2B | Figure 2—figure supplement 1B | WT | >0.9999 |
| Figure 2B | Figure 2—figure supplement 1B | *nhr-49(nr2041)* | 0.0037** |
| Figure 2B | Figure 2—figure supplement 1B | *hif-1(ia4)* | 0.0028** |
| Figure 2B | Figure 2—figure supplement 1B | *nhr-49(nr2041);hif-1(ia4)* | <0.0001**** |

All p-values are derived using ordinary two-way ANOVA corrected for multiple comparisons using the Tukey method. **p<0.01 and ****p<0.0001. WT = wild-type.
