# Supplementary material for: Nuclear hormone receptor NHR-49 acts in parallel with HIF-1 to promote hypoxia adaptation in Caenorhabditis elegans
Source: eLife. 2022 Mar 14;11:e67911. doi: 10.7554/eLife.67911 (PMC8959602; doi:10.7554/eLife.67911)
Supplement: Supplementary file 2. — Statistical comparison of each genotype’s ability to reach at least L4 stage from L1 stage following 48 hr exposure to 0.5% O2 as embryos compared to animals kept in 21% O2 for 48 hr. [file elife-67911-supp2.docx]

**Supplementary File 2.** **Statistical comparison of each genotype’s ability to reach at least L4 stage from L1 stage following 48 hr exposure to 0.5% O_2_ as embryos, compared to animals kept in 21% O_2_ for 48 hr.**

| **0.5% O_2_ Figure** | **21% O_2_ Figure** | **Genotype** | **p-value** |
| --- | --- | --- | --- |
| Figure 2B | Figure 2—figure supplement 1B | WT | >0.9999 |
| Figure 2B | Figure 2—figure supplement 1B | *nhr-49(nr2041)* | 0.0037** |
| Figure 2B | Figure 2—figure supplement 1B | *hif-1(ia4)* | 0.0028** |
| Figure 2B | Figure 2—figure supplement 1B | *nhr-49(nr2041);hif-1(ia4)* | <0.0001**** |

All p-values are derived using ordinary two-way ANOVA corrected for multiple comparisons using the Tukey method. **p<0.01 and ****p<0.0001. WT = wild-type.
